# Advice to remain active with arm pain reduces disability

**DOI:** 10.1093/occmed/kqad065

**Published:** 2023-06-01

**Authors:** K Walker-Bone, G J Macfarlane, K Burton, A M McConnachie, R Zhang, G T Jones

**Affiliations:** Monash Centre for Occupational and Environmental Health, University of Monash, Melbourne, Victoria, Australia; MRC Versus Arthritis Centre for Musculoskeletal Health and Work, MRC Lifecourse Epidemiology Centre, University of Southampton, Southampton, UK; MRC Versus Arthritis Centre for Musculoskeletal Health and Work, School of Medicine, Medical Sciences and Nutrition, University of Aberdeen, Aberdeen, UK; Aberdeen Centre for Arthritis and Musculoskeletal Health (Epidemiology Group), School of Medicine, Medical Sciences and Nutrition, University of Aberdeen, Aberdeen, UK; Department of Nursing and Midwifery, School of Human and Health Sciences, University of Huddersfield, Huddersfield, UK; Robertson Centre for Biostatistics, University of Glasgow, Glasgow, UK; Medical and Scientific Affairs, Astrazeneca UK Limited, 2 Pancras Square, 8th floor, London N1C 4AG, UK; MRC Versus Arthritis Centre for Musculoskeletal Health and Work, School of Medicine, Medical Sciences and Nutrition, University of Aberdeen, Aberdeen, UK; Aberdeen Centre for Arthritis and Musculoskeletal Health (Epidemiology Group), School of Medicine, Medical Sciences and Nutrition, University of Aberdeen, Aberdeen, UK

## Abstract

**Background:**

Arm pain is common amongst working-aged adults and causes substantial work disability. The results of a population-based randomized controlled trial (the ARM trial) suggested that advice to remain active reduced disability after 6 months.

**Aims:**

To verify ARM trial results amongst people in paid employment.

**Methods:**

The ARM trial recruited adults with distal arm pain referred for physiotherapy and randomized equally to three groups: wait-listed for physiotherapy (advised to rest); wait-listed for physiotherapy (advised to remain active) or early physiotherapy. The primary outcome was absence of disability at 26 weeks. Secondary analyses were undertaken amongst participants in paid employment.

**Results:**

Amongst 538 trial participants, 347 (64%) were in paid employment, mean age 46.1 years and 47% in manual work. Employed participants were randomized equally to the three arms. Amongst the 271 (78% workers with 26-week data), 43% of those advised to remain active were free from disability, as compared with 37% of those advised to rest. Forty per cent of those who waited for physiotherapy were disability-free as compared with 35% of those treated rapidly. Advice to rest was associated with lower chances of recovery amongst workers who lift/carry weights and those who believed work had caused their symptoms (*P* = 0.023).

**Conclusions:**

Although not powered as a trial for workers only, our findings suggest that advising activity was as beneficial for people currently in paid work and may be superior to advice to rest in reducing disability. Addressing harmful beliefs about causation of symptoms has the potential to reduce disability.

## Teaser

Arm pain is a common problem in the workplace. In contrast with the standard biomedical management of distal arm pain, these secondary analyses suggest that advising workers to keep using their arms as much as possible leads to less disability at 6 months, particularly amongst workers who lift heavy weights or believe work caused their symptoms. Early physiotherapy, however, made no difference to disability outcomes. Similar results were seen amongst workers diagnosed with lateral epicondylitis.

**Key learning points**

**What is already known about this subject:**

- Arm pain is common amongst workers and frequently disabling but optimal management is currently unknown.
- The results of a population-based randomized controlled trial (the ARM pain trial) suggested that advice to remain active was superior to advice to rest at reducing disability after 6 months.
- It is not known whether advice to remain active is as effective at reducing disability amongst workers doing manual jobs or physically demanding occupational activities.

**What this study adds:**

- Advice to remain active was as effective at reducing disability among workers and did not increase sickness absence or job loss.
- There was no advantage for disability at 6 months of early physiotherapy as compared with physiotherapy after waiting.
- The findings were unchanged no matter whether workers were doing manual jobs, performing a range of demanding occupational activities or had clinical findings of epicondylitis.

**What impact this may have on practice or policy:**

- Current information available on the NHS website has been updated to reduce the emphasis on advice to rest.
- Positive messages about using the arm and remaining at work result in less disability after 6 months.

## Introduction

An estimated 4.4 million UK working days were lost 2019/20 because of ‘work-related neck/upper limb disorders’ [1]. Indeed, upper limb disorders account for 45% of all occupational diseases in Europe [2]. Moreover, arm symptoms are frequently attributed to work [3] and have caused workplace epidemics [4] including ‘repetitive strain injury’ [5–7]. Some causes of arm pain have well-defined pathoanatomical features and diagnostic criteria (tenosynovitis), even if their aetiology is not fully understood but patients can present with pain and disability in the elbow, forearm wrist/hand without any distinct pathoanatomical features. Either way, rates of disability are high: amongst people consulting with pain affecting the elbow, forearm, wrist and/or hand in the community, 50% reported persistent symptoms 12 months later and 14% reported severely disabling symptoms [8].

Despite their frequency and impact, it is unknown how best to manage upper limb disorders. Injections, medication or physiotherapy might be effective in some conditions, but their clinical effect is inconsistent and any benefit from advice to rest the arm or avoidance of occupational activities has not been established [9]. Although psychosocial interventions, such as addressing health beliefs and encouraging activity and remaining at work, are potentially useful in the management of arm pain [9], their effectiveness in a working population remains to be determined. For these reasons, the ARM pain trial was funded [10]. This randomized controlled trial (RCT) recruited people with distal arm pain referred to physiotherapy and randomized to three groups: waiting-list physiotherapy and advice to rest; waiting-list physiotherapy and advice to remain active; or immediate physiotherapy. The main results [11] showed that, while awaiting physiotherapy, advice to remain active was superior to advice to rest in terms of reducing disability 26 weeks later, with no additional benefit of early physiotherapy. However, eligibility for the ARM trial was not based on work status. Given the frequency and impact of arm pain in the workplace, it is important to establish whether there is a different effect of advice to remain active amongst working trial participants, and also whether there are selective differences in response amongst people with varying physical work demands. Therefore, we re-analysed the ARM pain trial data to address these questions.

## Methods

The ARM trial protocol was registered (reference: ISRCTN79085082) [10] and the methods were reported elsewhere [11]. In brief, people with distal arm pain were recruited from 14 UK community clinics. Adults aged ≥18 years were eligible if they had been referred for physiotherapy with a new episode of distal arm pain (not received physiotherapy for arm pain within the past 12 months) but were excluded if the symptoms were thought to be referred (e.g. cervical); the pain was caused by a fracture, inflammatory rheumatic disease, cancer or complex regional pain syndrome; if there was a contraindication to remaining active; their referral was graded an ‘emergency’ and/or there was an ongoing medico-legal case. Eligible patients were identified from referrals and posted information about the trial. Interested patients were offered a pre-trial appointment to elicit written informed consent to participate. The baseline questionnaire collected demographic information; employment status; usual number of working days/hours; and workplace exposures including keyboard use >1 hour/day and >4 hours/day; wrist/finger movements >4 hours/day; repeated elbow movements >1 hour/day; vibration; working with arms above shoulder height >1 hour/day; exposure to heavy lifting (>5 kg or >10 kg) and exposure to pushing/pulling heavy weights. Questions were also asked about absenteeism, coping at work and beliefs about arm pain causation.

Participants were examined according to the Southampton examination protocol [12,13], including inspection, palpation, measurement of range of motion and standardized provocation tests. Staff at each recruitment site were taught the examination and were supported with a video and written instructions. A reliability assessment and refresher session were held mid-trial. Examination findings were analysed by a pre-defined computerized algorithm which assigned diagnostic labels to clusters of physical findings. For example, a diagnosis of lateral epicondylitis was assigned when pain and tenderness were elicited at the lateral epicondyle, and pain was provoked on resisted wrist extension.

Randomization was online (with telephone backup) allowing immediate allocation to a group, using a mixed randomization and minimization algorithm. This enabled balanced recruitment by recruitment site; site of symptoms (dominant, non-dominant or bilateral); hand/wrist versus elbow; and functional capacity defined by the modified Disabilities of the Arm, Shoulder and Hand (mDASH) score [8], using high, medium and low scores (0–5, 6–8 or 9–11).

The three trial groups were allocated to waiting-list physiotherapy plus a novel leaflet ‘Arm pain. How to deal with it - keep active to recover quickly’ [14] (arm pain is common, lasting damage is rare and recovery can be expected and explaining how to gradually increase activity and work participation); allocated to waiting-list physiotherapy plus an existing National Health Service leaflet: ‘Advice and Guidance on Arm Pain—Causes, Diagnosis, Treatment’ [14] (biomedical leaflet advocating rest and avoidance of activities that exacerbate symptoms); or early physiotherapy (usually within 1 week). The physiotherapy administered (whether early or after waiting) was not prescribed but all physiotherapists involved received pre-trial training at which current evidence underpinning treatment for specific and non-specific upper limb disorders was presented as interventions with some evidence of effect; interventions for which there was no evidence for/against benefit and interventions with evidence of harm [10]. Physiotherapy appointments were offered to everybody in the trial but those allocated to the waiting list were treated after a period which averaged 6–8 weeks depending upon the recruitment site.

The principal outcome measure was the reduction of disability as measured by the validated mDASH, which asks participants to report difficulties caused by an ‘ache or pain in the elbow, forearm, wrist or hand’ in the past 7 days, with any of a list of 11 separate activities (Options: yes, no, not applicable). The mDASH was assessed at baseline and by postal follow-up at 6-, 13- and 26-week post-randomization. Non-responders were telephoned to obtain verbal responses. Secondary outcomes were days of sickness absence at 6, 13 and 26 weeks; left job due to arm pain at 6, 13 and 26 weeks.

A mixed-effects logistic regression model was used to model the probability of achieving full recovery (mDASH = 0) and to estimate the difference in probability (rather than the odds ratios) between groups. The model included treatment group (as a three-level categorical variable), age, gender, study centre, pain location (elbow, wrist/hand or both), laterality (dominant, non-dominant or bilateral) and baseline function (mDASH: 0–5, 6–8 or 9–11). The rates of achievement of the primary outcome were compared firstly amongst working participants who received advice to remain active, as compared with those advised to rest. Subsequently, the effects of early physiotherapy versus physiotherapy after the usual waiting time were compared.

To explore whether there were differential responses by nature and/or type of work to the treatment received, a series of mixed-effects logistic models were constructed to estimate the probability of full recovery (mDASH = 0) at 26 weeks, with each model including treatment group, age, gender, pain location, laterality and baseline function. A separate model was constructed for manual versus non-manual work; keyboard use >4 hours/day; repetitive use of the fingers/wrist >1 hour/day; repetitive movements of the elbow >1 hour/day; vibration of the hand/arms; lifting/carrying weights >10 kg).

The trial was approved by the UK South Central (Hampshire A) Research Ethics Committee (reference: 11/SC/0107).

## Results

The target sample size was 555 (185 per group) estimating 90% power at 5% significance to detect an increase from 51% to 70% in the proportion free from disability at 26 weeks, allowing for 20% dropout [11]. The study recruited 538 participants amongst whom 347 (mean age 46 years, 175 men and 172 women) were in paid employment at least 20 hours/week with 47% working in a manual job. Table 1 summarizes the demographic and work characteristics of the employed participants, altogether and by treatment allocation. At baseline, 7% of workers reported sickness absence, 12% reported working fewer hours and a third reported having difficulties with some work tasks in the preceding week because of their arm pain. Baseline disability scores according to mDASH (median 6, IQR 3–8) were similar in the three groups.

**Table 1:** Demographic and work characteristics of employed participants in the ARM trial

		All workers (*n* = 347) (%)	Advice to remain active (*n* = 110)(%)	Advice to rest (*n* = 116) (%)	Early physiotherapy (*n* = 121)(%)
Age (years)	Mean (SD)	46.1 (11)	45.5 (11)	46.5 (11)	46.2 (10.0)
	Median (IQR)	47 (40–53)	46 (40–52)	46 (40–52)	47 (41–53)
Gender	Male	175 (50)	52 (47)	62 (53)	61 (50)
	Female	172 (50)	58 (53)	54 (47)	60 (50)
Handedness	Right	300 (87)	93 (85)	95 (82)	112 (93)
	Left	31 (9)	13 (12)	11 (10)	7 (6)
	Both	16 (5)	4 (4)	10 (9)	2 (2)
BMI (kg/m^2^)	Mean (SD)	27.3 (4.61)	27.1 (4.84)	27.5 (4.74)	27.1 (4.28)
	Median (IQR)	26.5 (24.0–29.7)	25.9 (24.0–30.2)	26.6 (23.6–29.6)	26.7 (24.2–29.8)
Employment status	Full time	271 (78)	79 (72)	94 (81)	98 (81)
	Part time	76 (22)	31 (28)	22 (19)	23 (19)
Type of work	Manual	160 (47)	49 (45)	63 (55)	48 (40)
	Non-manual	183 (53)	60 (55)	52 (45)	71 (60)
Physical work exposures	Average keyboard use >1 hour	205 (61)	64 (60)	62 (55)	79 (68)
	Average keyboard use >4 hours	153 (45)	49 (46)	51 (45)	53 (45)
	Other repetitive wrist/finger movements >4 hours/day	148 (48)	53 (54)	49 (48)	46 (45)
	Repeated movements of the elbow >1 hour/day	196 (58)	64 (60)	66 (58)	66 (55)
	Vibration of hands or arms	42 (12)	13 (12)	16 (14)	13 (11)
	Working with hands above shoulder height >1 hour/day	45 (13)	18 (17)	17 (15)	10 (9)
	Lifting/carrying weights >5 kg/day	137 (40)	42 (40)	46 (40)	49 (41)
	Lifting/carrying weights >10 kg/day	88 (26)	30 (28)	29 (25)	29 (24)
	Pushing/pulling heavy weights	109 (32)	35 (33)	37 (33)	37 (31)
No. (%) who took sick days in past week	Yes	26 (8)	8 (7)	10 (9)	8 (7)
Worked fewer hours past week	Yes	41 (12)	19 (17)	12 (10)	10 (9)
Difficulties performing normal work tasks past week	Yes	117 (34)	39 (36)	44 (39)	34 (29)
Baseline mDASH score	Mean (SD)	5.6 (2.69)	5.7 (2.59)	5.5 (2.79)	5.6 (2.71)
	Median (IQR)	6 (3–8)	6 (3–8)	6 (3–8)	5 (3–8)

**Figure 1.**
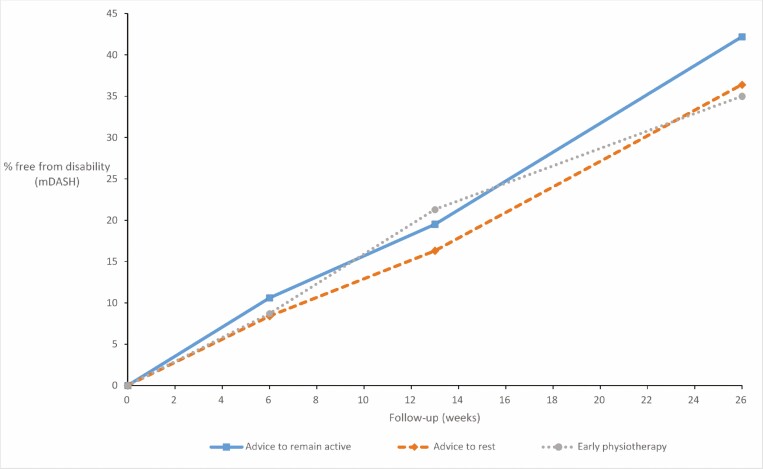
Proportion of workers free from disability according to mDASH at 6, 13 and 26 weeks in each arm.

At 26 weeks, 271 workers provided mDASH scores, at which time 35 (42%), 32 (36%) and 35 (35%) of workers in the advice to remain active, advice to rest and early physiotherapy groups, respectively, reported full recovery from disability in all 11 activities (mDASH = 0). Figure 1 summarizes the proportion of workers free from disability for 292 (84%), 272 (78%) and 271 (78%) of workers who provided data at each time point. There was a clear and steady improvement in all groups, with no evidence of a difference between groups in the proportion of participants achieving mDASH = 0 at 26 weeks (OR for advice to rest versus advice to remain active 0.78, 95% CI 0.40–1.51; and OR for odds of mDASH = 0 for early physiotherapy versus physiotherapy after waiting 0.68, 95% CI 0.35–1.30).

The series of mixed-effects models showed no difference in the odds of achieving recovery at 26 weeks when comparing those who were, with those who were not, doing: manual versus non-manual work; keyboard use >4 hours/day; repetitive movements of the wrist/fingers >1 hour/day; repetitive elbow movements >1 hour/day; vibration of the hands/arms and lifting/carrying weights >10 kg (data not shown). Likewise, there were no differences in sickness absence reported at 6, 13 or 26 weeks or in rates of leaving work amongst people in the three groups no matter whether they were doing a manual job or physically demanding occupational activities.

Table 2 summarizes the odds (95% CIs) for recovery at 26 weeks amongst working participants by treatment allocation and work exposures and beliefs about causation. Advice to rest tended to be associated with lower odds of recovery amongst people exposed to physically demanding activities but was only found significantly associated amongst those who carried/lifted weights >10 kg in an average working day (OR 0.13, 95% CI 0.03–0.57). Likewise, people who believed that work caused their symptoms appeared less likely to be free of disability at 26 weeks when advised to rest (OR 0.25, 95% CI 0.08–0.83).

**Table 2.** Impact of treatment on odds of recovery at 26 weeks (mDASH = 0) comparing response by mechanical and psychosocial work characteristics for the employed participants in the ARM trial

Type of work or exposure at work		Advice to remain active versus advice to rest			Early physiotherapy versus physiotherapy after time on waiting-list		
		OR	95% CI	*P* value	OR	95% CI	*P* value
Manual work	Yes	0.43	0.16–1.14	0.090	0.36	0.13–0.97	0.043
	No	1.21	0.48–3.09	0.684	1.06	0.45–2.51	0.892
Keyboard use >4 hours/day	Yes	0.63	0.24–1.62	0.333	0.57	0.22–1.45	0.237
	No	0.82	0.32–2.11	0.682	0.73	0.30–1.81	0.502
Other repetitive movements fingers/wrist >1 hour/day	Yes	0.50	0.17–1.44	0.197	0.60	0.22–1.63	0.319
	No	1.00	0.37–2.73	0.994	0.58	0.22–1.51	0.262
Repetitive movements of the elbow >1 hour/day	Yes	0.68	0.28–1.63	0.385	0.40	0.16–0.96	0.041
	No	0.76	0.28–2.08	0.588	1.21	0.45–3.21	0.706
Vibration of hands/arms	Yes	0.37	0.06–2.34	0.287	0.24	0.04–1.50	0.126
	No	0.78	0.38–1.59	0.492	0.76	0.38–1.52	0.435
Lifting/carrying heavy weights >10 kg	Yes	0.13	0.03–0.57	0.007	0.26	0.07–0.95	0.042
	No	1.19	0.55–2.60	0.657	0.90	0.41–1.96	0.792
Belief that work causes symptoms	Yes	0.25	0.08–0.83	0.023	0.65	0.25–1.69	0.372
	No	1.21	0.50–2.89	0.674	0.65	0.27–1.54	0.324
Belief that work makes symptoms worse	Yes	0.72	0.34–1.50	0.378	0.59	0.28–1.23	0.161
	No	0.78	0.18–3.41	0.740	0.77	0.19–3.18	0.719

The mixed-effects model including 82 workers with epicondylitis (yes versus no) found no difference in odds of recovery (mDASH = 0) at 26 weeks for those with epicondylitis as compared with those without (OR 1.01, 95% CI 0.59–1.73, *P* = 0.968).

## Discussion

Among workers with distal arm pain, advice to remain active was associated with less disability than advice to rest at 26 weeks. There was no evidence of improvement in the odds of recovery conveyed by early, as compared with delayed (usual waiting time) physiotherapy. There was no evidence of differential response in the three groups amongst people doing manual versus non-manual work, or exposed versus not exposed to a range of physically demanding activities nor were there differences in freedom from disability or sickness absence at 6, 13 or 26 weeks. However, there was some evidence amongst workers who lift/carry weights >10 kg that advice to rest while awaiting physiotherapy led to poorer chances of achieving recovery at 26 weeks (0.13, 95% CI 0.03–0.57). Furthermore, amongst 82 workers with clinical epicondylitis, we found no evidence of poorer outcomes in terms of disability or sickness absence from advice to remain active.

Some limitations must be considered. First, the ARM trial was powered on the assumption that 50% of participants would achieve mDASH = 0 by 26 weeks and assuming a 20% improvement with advice to remain active. When fewer than 50% of participants achieved mDASH = 0, a re-estimation of the power suggested that the main trial had 89% to detect an increase in recovery from 32% to 52% [11]. However, the current analyses are confined to the 78% of participants working >20 hours/week and therefore may be underpowered to show a difference by group. The aim of these secondary analyses was to investigate whether there was any evidence of poorer outcomes amongst people in manual jobs or jobs involving physically demanding exposures given advice to remain active: these results provide some reassurance, although we cannot rule out being underpowered to find a small adverse effect (unlikely in that we found no adverse effect, even non-significant). Second, although these are intention-to-treat analyses, it is important to note that complete outcome information was not obtained for 22% of workers in the trial, despite follow-up telephone calls. Non-response rates were slightly higher in the workers than those achieved overall (19%), suggesting perhaps that some of the workers (and perhaps those least functionally impaired) returned to work and therefore became unavailable for research. Third, the occupational exposures (and manual versus non-manual classification) reported in these analyses were self-reported. The validity of self-reported upper limb exposures has been questioned [15] but, although not perfect, significant relationships have been found between self-reported ratings of force, pace and wrist motion when compared with measured physical exposures and, moreover, workers with hand/wrist symptoms were found to be more accurate in estimating exposures than those without [16]. As this trial was recruited from 14 UK sites, objective assessment of upper limb exposures was not feasible. Importantly, any effect of use of self-reported measures should not have led to differential classification of occupational activities between the three intervention arms after randomization (the randomization algorithm did not take into account of the type of work), so any misclassification should have been evenly distributed and not obscured any real adverse effect of advice to remain active. Fourth, the effects of any work-based adjustments that might have been put in place to accommodate workers with distal arm pain could not be considered. That said, the RCT design should mean that workers with/without adjustments should be equally spread amongst the three arms of the trial. Finally, this trial was designed with a 6-month primary outcome based on epidemiological studies but consequently, the data allow no further conjecture about disability amongst participants beyond 6 months.

In the 1990s, the paradigm for the management of low back pain changed substantially [17,18], based on strong evidence that the traditional medical model of management was increasing the risk of disability. Although disability from low back pain is not reducing globally, it is increasing most in low- and middle-income countries [19]. Although a nociceptive source is rarely identified for back pain, pain and disability are thought to be dependent upon genetic, psychological and social factors (more disability with poorer socio-economic status), biophysical factors, comorbidities, and pain-processing mechanisms. Distal arm pain shares risk factors with low back pain, also lacks a nociceptive cause in most cases and can be associated with severe disability, including for work [20]. As with back pain [21], our results, like those of others [22], suggest that harmful beliefs can be importantly associated with the risk of disability. We found that believing arm pain was caused by work was associated with poorer chances of recovery at 26 weeks when advised to rest. This is particularly important, given that manual handling regulations and other health and safety-related guidance generally reinforce the belief that work causes upper limb pain [20]. It may be that the traditional medical model leaflet encouraged or strengthened this adverse belief, or created kinesophobia which prevented recovery but, either way, it would seem that there is no added harm from a de-medicalising approach based on biopsychosocial factors. Based on our findings, and their congruence with those reported for low back pain, it would seem appropriate to change the standard medical information being provided to people with arm pain.

That early physiotherapy was not better than physiotherapy after a waiting period was perhaps surprising. For the most part, early intervention is recommended amongst people with musculoskeletal conditions, not least because the longer duration of sickness absence is associated with reducing odds of ever returning to work [23]. However, there is already rather limited evidence of the effectiveness of physiotherapy for distal arm pain, whether caused by specific [24] or non-specific disorders [25]. In UK clinics, physiotherapists do not usually offer corticosteroid injections, for which there is some limited evidence for a short-term benefit in conditions such as epicondylitis [26], de Quervain’s tenosynovitis [27] or trigger finger [28], so the treatment offered would include splints, manual therapies, massage or exercises. Whilst the effectiveness of these approaches is possibly limited, one benefit of early physiotherapy would be to have a trusted healthcare professional reinforce healthy beliefs, encourage movement and de-medicalize the symptoms. Physiotherapists in this trial were not trained to do this in the absence of evidence for this approach but were encouraged to assess and treat each patient according to best practice. Analysis of the actual treatment offered showed no differences between the three groups, so our findings would suggest that either physiotherapists do not emphasize these messages or that people with arm pain are no more likely to take such advice on board whether given this information early or some weeks later.

There is an established evidence base for an increased risk of epicondylitis in association with physically demanding activities of the upper limb, for example, in the meat processing industry [29]. Consequently, the recommendation has been that the management of epicondylitis should involve resting the elbow and avoidance of provoking occupational activities [30]. As a significant proportion of the working ARM trial participants had signs and symptoms consistent with this diagnosis, we analysed the response to advice to remain active in this sub-group. Our finding that there was no suggestion of a poorer response is encouraging, although must be treated with caution given that the statistical power may have been insufficient to show an effect. If this finding can be replicated, it suggests that de-medicalization and remaining active may be a more effective approach to reducing disability for specific, as well as non-specific, causes of upper limb pain.

These secondary analyses in the ARM trial suggest that advice to remain active was as effective at reducing disability among workers and did not increase sickness absence or job loss. In particular, workers who believed their pain was caused by work and those who carried/lifted weights at work were less likely to have recovered at 6 months when advised to rest. There was also no evidence that early physiotherapy reduced disability among workers compared with physiotherapy after waiting 6–8 weeks.
